# A de novo dominant mutation in KIF1A associated with axonal neuropathy, spasticity and autism spectrum disorder

**DOI:** 10.1111/jns.12235

**Published:** 2017-09-11

**Authors:** Pedro J. Tomaselli, Alexander M. Rossor, Alejandro Horga, Matilde Laura, Julian C. Blake, Henry Houlden, Mary M. Reilly

**Affiliations:** ^1^ MRC Centre for Neuromuscular Diseases and National Hospital for Neurology and Neurosurgery UCL Institute of Neurology London UK; ^2^ Department of Neuromuscular Disorders, Clinical Hospital of Ribeirão Preto University of São Paulo Ribeirão Preto SP Brazil; ^3^ Department of Neurogenetics The National Hospital for Neurology and Neurosurgery, UCL Institute of Neurology London UK

**Keywords:** autism spectrum disorder, KIF1A, Kinesin family member 1A gene, next‐generation sequencing, peripheral neuropathy

## Abstract

Mutations in the kinesin family member 1A (KIF1A) gene have been associated with a wide range of phenotypes including recessive mutations causing hereditary sensory neuropathy and hereditary spastic paraplegia and de novo dominant mutations causing a more complex neurological disorder affecting both the central and peripheral nervous system. We identified by exome sequencing a de novo dominant missense variant, (c.38G>A, p.R13H), within an ATP binding site of the kinesin motor domain in a patient manifesting a complex phenotype characterized by autism spectrum disorder (ASD), spastic paraplegia and axonal neuropathy. The presence of ASD distinguishes this case from previously reported patients with de novo dominant mutations in KIF1A.

## Introduction

Recessive mutations in the gene kinesin family member 1A (*KIF1A*) have been reported as causative of hereditary sensory neuropathy type 2C (OMIM: 614213) and hereditary spastic paraplegia type 30 (OMIM: 610357) *(Riviere et al.,* 2011 *; Klebe et al.,* 2012 *)*. *KIF1A* is a member of the kinesin family and functions as an anterograde motor protein that transports membranous organelles along microtubules. More recently, *de novo* dominant mutations have been shown to cause a complex phenotype characterized by developmental delay, cerebellar ataxia, spasticity, and peripheral neuropathy *(Okamoto et al.,* 2014 *; Esmaeeli Nieh et al.,* 2015 *; Ohba et al.,* 2015 *)*. Autism spectrum disorder (ASD) is a neurodevelopmental disorder characterized by deficits in social interaction, language and communication, and the presence of repetitive or unusual behaviors *(Levy et al.,* 2009 *)*. We report a patient with a *de novo* missense variant in *KIF1A* presenting with a complex phenotype characterized by ASD, axonal neuropathy, and spasticity. This case further expands the phenotype of dominant mutations in *K1F1A*.

## Case Report

A 20‐year‐old male was seen with early onset spasticity, axonal neuropathy, and ASD. He is the second child of unrelated and healthy parents. He was born at term but his developmental motor milestones were delayed. At 3 years of age, he was noted to interact poorly with other children. After starting main stream school, he was diagnosed with attention deficit and hyperactivity disorder (ADHD) and displayed significant difficulties with written and spoken language and was given a formal diagnosis of ASD. Additional features of spasticity and neuropathy became evident in adulthood. Neurological examination at age 20 demonstrated normal cranial nerves with a positive jaw jerk and pout reflex. The upper limbs were normal. He had a spastic gait with in‐turning of the feet, *pes cavus*, and mild wasting of the intrinsic foot muscles but only minimal weakness of ankle dorsiflexion. Sphincter function was normal. Deep tendon reflexes were normal in the upper limbs, but pathologically brisk in the lower limbs with extensor plantar responses. Sensory examination revealed reduced vibration sensation to the ankles but was otherwise normal. Nerve conduction study at age 18 revealed a length‐dependent sensory and motor axonal neuropathy with signs of chronic denervation in the lower limbs (Table 1).

**Table 1:** Nerve conduction study (aged 18).

Sensory NCS	Right		Left	
	Amplitude (μV)	Conduction velocity (m/s)	Amplitude (μV)	Conduction velocity (m/s)
Median	10	50	11	49
Ulnar	7	52	5	48
Radial	11	55	11	58
Sural	Absent	—	Absent	—
Superficial Peroneal	Absent	—	Absent	—

Motor NCS	Latency (ms)	Amplitude (mV)	Conduction velocity (m/s)	F wave (ms)
Right median (APB)	3.6	9.7	50	30
Right ulnar (ADM)	3.0	8.3	51	31
Right common peroneal (EDB)	Absent	—	—	—
Right posterior tibial (AHL)	5.0	0.3	34	Absent

ADM, abductor digiti minimi; AHL, abductor halluces; APB, abductor pollicis brevis; EDB, extensor digitorum brevis; NCS, nerve conduction study.

We performed whole‐exome sequencing in the proband to determine the causative gene as previously described using the Illumina Nextera rapid capture focused enrichment kit and run on the Illumina HiSeq 2500 *(Cottenie et al.,* 2014 *; Shigemizu et al.,* 2015 *)*.

**Figure 1:**
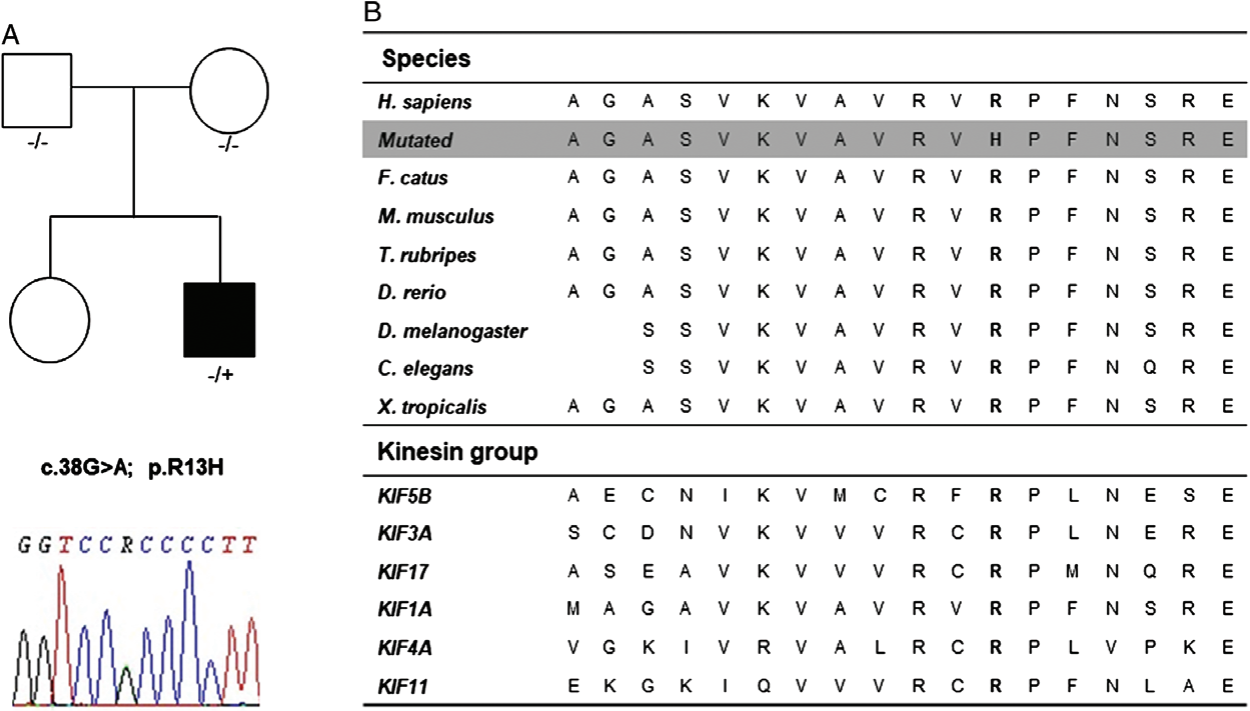
Panel (A) shows the pedigree and chromatograms of the proband. Panel (B) shows that the arginine at position 13 of KIF1A is conserved from man to Xenopus. tropicalis and is also conserved across other kinesin proteins.

Next‐generation sequencing exome analyses revealed 21,683 variants within coding regions or splicing sites. Variants were filtered for non‐synonymous and nonsense variants with a minor allele frequency <0.5% in the The Exome Aggregation Consortium (ExAC), 1.000 Genomes and Exoma Variant Server (EVS) public databases, and for sequence quality (read depth > 7), resulting in 420 variants. Additional filtering was performed for genes associated with neurological diseases and seven variants were identified. One heterozygous variant was detected in *DHTKD1*, a gene associated with autosomal dominant CMT2Q but did not segregate with the disease. Three heterozygous variants in genes associated with autosomal recessive disorders (*LYST*, *PLEKHG5* and *NEB*) were identified. Mutations in two additional genes associated with distal myopathy and deafness (*FLNC* and *GJB3*) were identified but were predicted benign using *in silico* analysis. A novel missense mutation, c.38G>A; p.R13H in *K1F1A* was identified (RefSeq assessing number NM_001244008.1). This mutation is located within a highly evolutionary conserved region both between species and also among different kinesin proteins (1 [KIF5B], 2 [KIF3A and KIF17], 3 [KIF1A], 4 [KIF4A], and 5 [KIF11]) where it forms part of the ATP binding site (Fig. 1). Sanger sequencing of the proband and parent's DNA was performed as previously described *(Liu et al.,* 2014 *)*, and confirmed the mutation had arisen *de novo* in the proband. The mutation resides in the motor domain of *KIF1A*, providing further evidence in support of the pathogenicity of this novel mutation. *In silico* analysis using MutationTaster, Polyphen‐2, and SIFT predicted the p.R13H mutation to be pathogenic. This variant was not present in the Single Nucleotide Polymorphism database (dbSNP), EVS, NHLBI Exome Sequencing Project, 1000 Genomes and ExAC databases. There were no additional variants in known ASD‐related genes.

## Discussion

We describe a novel *de novo* dominant mutation in the kinesin motor domain of *KIF1A* as a cause of a complex neurodevelopmental disorder characterized by ASD, spasticity and axonal neuropathy. ASD is a complex and poorly understood condition, and approximately 10%–15% of cases are associated with mutations in single genes *(Levy et al.,* 2009 *)*. Several genes (e.g., FMR1, MECP2, NLGN4X, and NLGN3) have been implicated in ASD but none have been reported in association with hereditary spastic paraplegia or axonal neuropathy.

*KIF1A* encodes a neuron‐specific ATP‐dependent motor protein which is important for anterograde axonal transport of cargo including synaptic vesicle precursors *(Okada et al.,* 1995 *; Lee et al.,* 2003 *)*, as well as post‐synaptic proteins involved in synaptic plasticity *(Lee et al.,* 2015 *)*. To date, recessive nonsense mutations have been reported as causative of hereditary sensory neuropathy with or without minimal intellectual disability whereas recessive missense mutations in the motor domain are causative of hereditary spastic paraplegia. *De novo* dominant mutations within the motor domain have been reported in association with complex phenotypes characterized by intellectual disability and the variable presence of cerebellar atrophy, spastic paraparesis, optic nerve atrophy, peripheral neuropathy, and epilepsy *(Lee et al.,* 2015 *)*.

The p.R13H mutation is located in a highly conserved residue within the ATP binding site, a region necessary for the hydrolysis of ATP required for force generation and vesicle transport along microtubules. *In utero*, this mutation may affect cortical neuronal migration resulting in neurodevelopmental disorders such as ASD. The p.S69L mutation in *KIF1A* has previously been reported in a family with pure spastic paraplegia and in whom the proband but not the affected father had attention deficit disorder. The authors concluded it was not part of the phenotype as it did not segregate with the mutation *(Ylikallio et al.,* 2015 *)*, however, the presence of ASD in our case suggests that it was probably part of the disorder. Mutations in the retrograde transport protein, *DYNC1H1*, have already been reported as a cause of ADHD, cortical migration defects and motor neuropathy further highlighting the importance of motor proteins in cortical and motor neuron development *(Scoto et al.,* 2015 *)*. This case report suggests that ASD and ADHD should be considered within the phenotypic spectrum of dominant mutations in KIF1A and further expands the spectrum of phenotypes associated with *KIF1A* mutations.
